# The value of Interferon β in multiple sclerosis and novel opportunities for its anti-viral activity: a narrative literature review

**DOI:** 10.3389/fimmu.2023.1161849

**Published:** 2023-06-02

**Authors:** Gianmarco Bellucci, Angela Albanese, Caterina Rizzi, Virginia Rinaldi, Marco Salvetti, Giovanni Ristori

**Affiliations:** ^1^ Department of Neurosciences, Mental Health and Sensory Organs, Centre for Experimental Neurological Therapies (CENTERS), Sapienza University of Rome, Rome, Italy; ^2^ Merck Serono S.p.A., An Affiliate of Merck KGaA, Rome, Italy; ^3^ Department of Health Sciences, University of Genoa, Genoa, Italy; ^4^ Istituti di Ricovero e Cura a Carattere Scientifico (IRCCS) Istituto Neurologico Mediterraneo Neuromed, Pozzilli, Italy; ^5^ Neuroimmunology Unit, Istituti di Ricovero e Cura a Carattere Scientifico (IRCCS) Fondazione Santa Lucia, Rome, Italy

**Keywords:** multiple Sclerosis, interferon β, SARS-CoV-2, Epstein-Barr virus, immune cells

## Abstract

Interferon-beta (IFN-β) for Multiple Sclerosis (MS) is turning 30. The COVID-19 pandemic rejuvenated the interest in interferon biology in health and disease, opening translational opportunities beyond neuroinflammation. The antiviral properties of this molecule are in accord with the hypothesis of a viral etiology of MS, for which a credible culprit has been identified in the Epstein-Barr Virus. Likely, IFNs are crucial in the acute phase of SARS-CoV-2 infection, as demonstrated by inherited and acquired impairments of the interferon response that predispose to a severe COVID-19 course. Accordingly, IFN-β exerted protection against SARS-CoV-2 in people with MS (pwMS). In this viewpoint, we summarize the evidence on IFN-β mechanisms of action in MS with a focus on its antiviral properties, especially against EBV. We synopsize the role of IFNs in COVID-19 and the opportunities and challenges of IFN-β usage for this condition. Finally, we leverage the lessons learned in the pandemic to suggest a role of IFN-β in long-COVID-19 and in special MS subpopulations.

## Introduction

Multiple Sclerosis (MS) is a chronic demyelinating disease in which inflammation and neurodegeneration cause accumulating central nervous system (CNS) damage and clinical disability. It is a multifactorial disorder with a complex etiology and pathophysiology: multiple genetic and environmental factors interact to establish and maintain a dysimmune attack, which halts neural homeostasis (1). Among environmental factors, the causal role of the Epstein-Barr Virus (EBV) is getting increasing support: from both large seroepidemiological studies (2) indicating that EBV infection is necessary for and precedes disease onset, and experimental evidence of biological mechanisms involving EBV in disease processes (3, 4).

The COVID-19 pandemic challenged researchers in a rush for actionable evidence on viral infections biology, to rapidly reach the goal of efficient therapeutic and preventive strategies against SARS-CoV-2. Lots of work focused on managing people at risk for severe forms of COVID-19, including “fragile” people suffering from neurological disorders and immune-suppressed people. Since the beginning of the pandemic, there has been a special interest in the implications of COVID-19 in people with MS, which are being treated with disease-modifying therapies (DMTs) that impact the immune system.

The largest studies showed a similar incidence of COVID-19 in people with MS (pwMS) compared with the general population, but a higher risk for severe course (ICU admission and death), that correlated with age, higher EDSS, comorbidities and treatment with anti-CD20 monoclonal antibodies (5–9).

Meanwhile, most studies highlighted a protective effect of IFN formulations (8, 10, 11). This fact was traced back to the biology of these endogenous antivirals – which are the forerunners of MS DMTs, having been approved almost thirty years ago (12) – linked to the hypothesis of a viral etiology of MS.

In this review, we discuss the value of IFN-β in MS through the lens of its antiviral properties. We then point out analogies and differences between possible activities against EBV and SARS-CoV-2, considering the data on IFN-β repositioning in COVID-19. Finally, we outline the potential uses of this molecule for selected MS patients and beyond.

## Methods

A comprehensive literature search was performed between April 1, 2022 and April, 15 2023 in the PubMed database with the following terms: “Interferon”, “Interferon β” or “β interferon”, “multiple sclerosis”, “Covid-19” or “SARS-CoV-2”, “Epstein Barr Virus” or “EBV”. We retrieved articles published in English; only papers with full text available were included. The reference lists of the selected articles and the relevant links were also manually reviewed for additional eligible papers.

The inclusion criteria for article selection were to include papers regarding: the role of viral infections in MS etiology, biological activities of IFNs in viral infections, the MoA of IFN-β in MS, in COVID-19 and in the context of EBV biology.

## Biology and mechanism of action of IFN-β in multiple sclerosis

IFNs are classified into three groups (type I, II, III) based on their structure, origin, cellular receptors and final effects. Interferon-β belongs to the type I IFN family, like IFN-α, and 15 other molecules which constitute a major line of host defense against viruses and other microorganisms. IFN-γ is the only member of the type II family; IFN-λ1 to λ4 constitute the recently discovered type III group.

Almost all cells in the body can produce type I IFNs; however, the greatest amount derives from plasmacytoid dendritic cells (pDCs), lymphocytes, and a few non-immune cells (such as fibroblasts and endothelial cells). Two patterns of IFN-I production can be distinguished: (a) a physiological, tonic, low-dose constitutive production, aiding in immune homeostasis; (b) a high-dose, phasic secretion, mainly by the innate cells, that follows the interaction of pathogen-associated molecular patterns (PAMPS) with pattern recognition receptors (PRRs) (13). Indeed, “IFNs” were named by Isaacs and Lindemann (14) due to their capacity to interfere with viral replication.

The binding of secreted type I IFNs with membrane IFNAR1/2 receptors induces the JAK-STAT pathway activation, leading to the nuclear translocation of IRF9, which guides the regulation of the so-called interferon-stimulated genes (ISGs) (15) whose products orchestrate an antiviral state in bystander cells and tackle the replication cycle in infected cells.

Furthermore, IFN-β exerts broad immune-modulating activities. It has been shown to reduce the expression of Th1 cytokines (TNFα, LTα, IFN) and MCH-II molecules on antigen-presenting cells (APCs), thus diminishing lymphocyte activation, while favoring an anti-inflammatory setup through the secretion of IL4, IL10 and TGF-β. These cytokines decrease the expression of matrix metalloproteinases (MMPs), adhesion molecules (VLA-4) and endothelial vascular cell adhesion protein (VCAM), lessening the ability of lymphocytes and monocytes to infiltrate tissues, including the CNS through the blood-brain barrier (BBB) (12). The effect on B lymphocytes is mediated by the augmented expression of apoptotic markers like Annexin-V and active caspase-3, promoting the death of memory B cells (16). This pathway may be altered in MS, whereby a pro-survival status of B cells was reported to be due to down-regulation of the interferon response factor (IRF) 1 and C-X-C motif chemokine 10 axis (17).

These wide-acting, modulating effects are thought to be the main mechanism of action (MoA) of IFN-β in MS treatment. Since a low serum level of IFN-β was found in the majority of MS patients (18), its therapeutic supplementation could serve as a rescue of this defect. A profound dysregulation of ISG transcription has been confirmed in both MS and its experimental models, more markedly in B cells (19, 20). This signature may reflect an MS-specific genetic predisposition: indeed, an enrichment of type I IFN signaling and antiviral pathways were found in the SNPs associated with disease development by several GWAS (21, 22).

Accordingly, the antiviral action of IFN-β could contribute to its efficacy in MS. The hypothesis of a viral etiology of MS stands on solid epidemiological, clinical and experimental evidence linking primarily EBV (but also HHV6 among Herpesviridae, and human endogenous retroviruses, HERVs) to disease development and progression (2–4, 23). This evidence and the above-mentioned immunomodulatory actions of IFNs may contribute to explain its beneficial effects as a therapeutic approach in MS.

## Antiviral effects against Epstein-Barr virus

EBV is a herpesvirus infecting 95% of individuals worldwide and >99% of people with MS. Following the infection, which is usually asymptomatic or can lead to infectious mononucleosis, the virus establishes a lifelong latency in human B cells, with sporadic reactivations and lytic replication (24).

Several mechanisms have been proposed through which EBV initiates and sustains MS pathogenic processes. EBV infection may unleash autoimmune clones through molecular mimicry between viral proteins and CNS antigens (3, 25, 26). EBV-infected memory B cells in the periphery may activate T cells to enter the CNS (27), also by producing an EBV-encoded interleukin-10-like protein that counteracts the human immunomodulatory IL-10 (28). Inadequate CD8+ killing of EBV-infected cells may result in the accumulation of EBV-infected autoreactive B cells in the CNS leptomeningeal follicles (29). There, EBV-infected B cells may fuel CNS-compartmentalized inflammation and neurodegeneration through cytotoxic bystander injury, interaction with self-reactive T cells and autoantibody production (30). Furthermore, broad effects on the immune response may arise from host-viral interactions involved in genetic regulation (31), influenced by both the host’s and viral genetic asset (32, 33). EBV may institute pathological synergies with MS-associated Human-Herpesvirus 6 (HHV6) and human-endogenous retroviruses, contributing to a neurotoxic antiviral response (34, 35). Additionally, EBV reactivation has been linked to relapse triggering: antibodies targeting EBV early antigen (EA), which is expressed during the lytic phase, and EBV-DNA have been detected during clinical and radiological MS activity (36–38).

At first, when EBV attacks the host’s cells, viral lipids and proteins are sensed by transmembrane TRL2; viral DNA and transcribed RNA are recognized in endosomes by TRL7 and TRL9; viral RNA also binds RIG-I-like receptors triggering a robust IFN production. However, as for other pathogens, EBV has evolved strategies to evade and counteract the antiviral IFN response, extensively reviewed in Lange et al. (39). Sensors downregulation and inhibition is mostly operated by BGLF5, BPLF1, LMP-1 and the miR-BART6-3p. Kinase cascade signaling is counteracted by EBNA1, LMP-1 and LMP2; then, IRFs, which induce genetic transcription of IFN genes, are tackled at various levels by BGLF4, BFRF1, BRLF1 and BZLF1 (40–43). The result is both a reduced IFN production and an impaired ISG activity that favor the establishment and persistence of viral latency, as well as viral replication during reactivation.

IFN-β administration could therefore counterweigh such signature at multiple points. A deficit in the TRL7-induced pathway has been found in B cells derived from pwMS, which is reverted *in vivo* by IFN-β administration (44). Severa and colleagues showed that the *in vitro* exposure to IFN-β rescues the IFN pathway defects in B cells derived from MS patients, sustaining antiviral processes, leading to a reduction of EBV-infected and proliferating B cells (19). This data mirrors the evidence of a reduction in pathogenic, memory B cells in the blood of pwMS receiving IFN-β (16) suggesting that both the interference with viral machinery and the depletion of viral reservoir mediate the anti-EBV activity of IFN-β.

Virus-specific CD8+ T cell responses play a pivotal role in the control of EBV infection (45): if quantitatively or functionally deficient, EBV reactivation would be unrestrained; an excessive cellular activation during the lytic phase would cause immunopathology. In MS, such dysregulation is hypothesized to happen within the CNS, whereby latently infected B cells would establish a “sanctuary” evading the virus-specific response; viral reactivation would trigger an enhanced immune activation, with bystander damage of the CNS (46). Indeed, a high frequency of CD8+ T cells specific for lytic EBV antigens has been identified in the peripheral blood of pwMS with active disease (47) which is reduced by IFN-β. A similar effect was found for CD4+ T cell responses against EBNA-1 (48).

**Figure 1:**
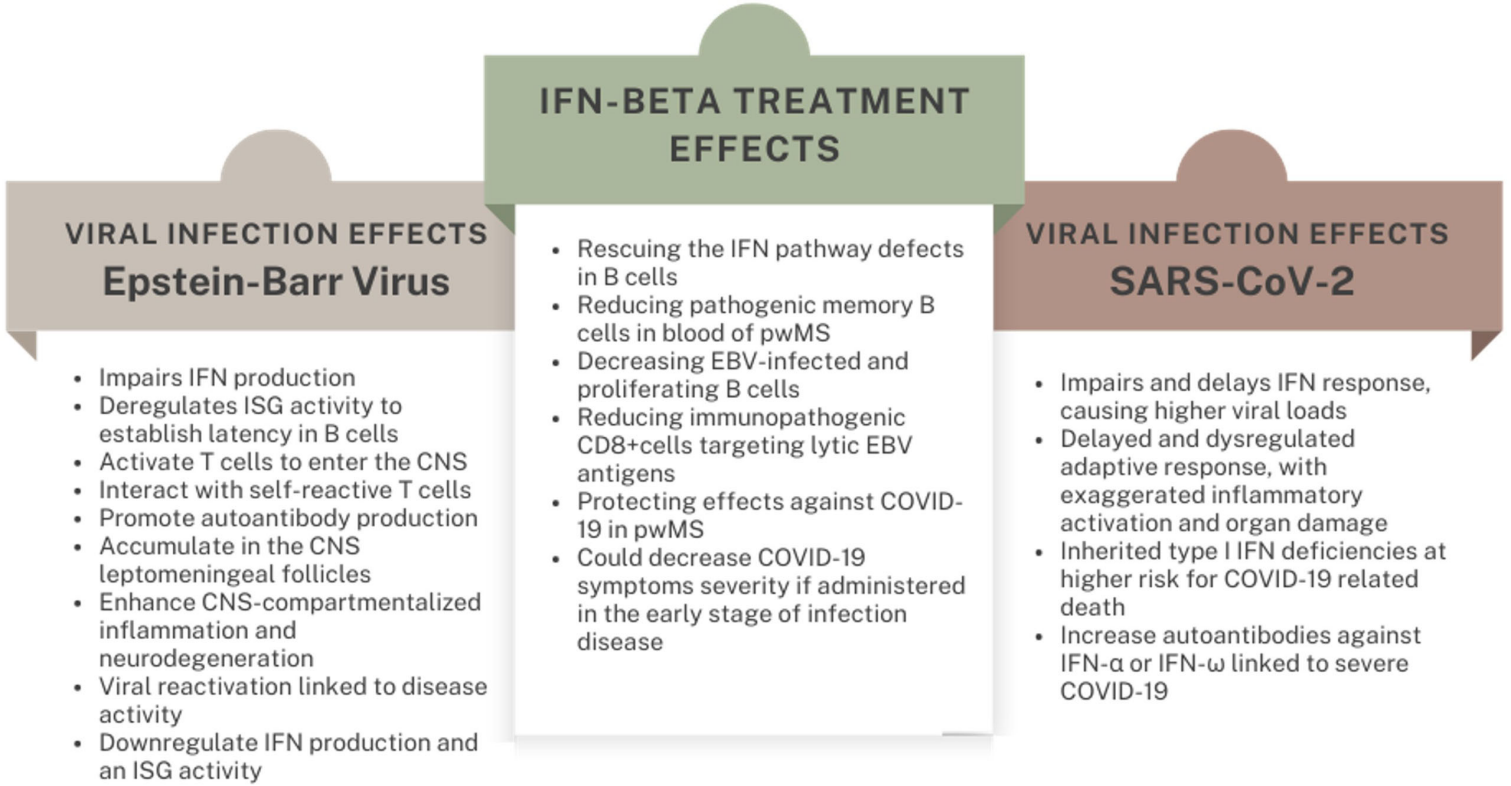
Immunological effects of viral infections and related IFN-β treatment outcomes in pwMS.

Overall, these data suggest a scenario in which the efficacy of IFN-β in the treatment of MS rests on wide immunomodulatory effects resulting, at least in part, from its antiviral properties (**Figure 1**).

## Antiviral effects against SARS-CoV-2

As of September 2022, SARS-CoV-2, the causative agent of COVID-19, has led to more than 6.5 million deaths worldwide. The novel virus emerged in late 2019 evolving from the clades of Sarbecoviruses, which also includes SARS-CoV (49). COVID-19 is a multifaceted disease, with primary respiratory involvement and systemic consequences, whose severity depends on a dysregulated host’s immune response to the viral infection: a delayed or impaired IFN response, favoring viral replication and diffusion, and an exaggerated inflammatory activation, leading to immunopathology (50).

The induction of IFN response by SARS-CoV-2 is started through TRL3 and TLR7-mediated sensing of viral RNAs in endosomes and by MDA5 in the cytosol (51, 52) initiating MAVs-IRFs cascade and type I and III IFN production.

Similar to other Coronaviruses, SARS-CoV-2 leverages several mechanisms to hijack IFN response (53, 54). IFN-I production is antagonized by NSP6 and NSP13, which limit TBK1 action on IRF3, and ORF6, which impedes IRF3 translocation into the nucleus.

Additionally, IFN-I signaling is tackled by NSP1, NSP6, NSP13, ORF6, ORF7a, and ORF7b by interfering with STAT1/STAT2 phosphorylation and consequent nuclear translocation with IRF9 (53, 55); meanwhile, the structural Membrane (M) protein suppresses the expression of IFN-β and ISGs interfering with RIG-I/MDA5/IKKϵ/TBK1 signaling (56). The ancestral strains of SARS-CoV-2 were far more sensitive to IFNs than SARS-CoV (57). Nonetheless, nowadays IFN evasion is among the factors guiding the emergence and selection of SARS-CoV-2 variants of concerns. A systematic *in vitro* screening of antiviral activities of 17 IFN formulations against 5 SARS-CoV-2 strains revealed a trend of increasing (17 to 122-fold) IFN resistance of evolving variants, with the B.1.1.7 strain exhibiting the highest IFN-I antagonism. Furthermore, this study highlighted IFN-alpha-8 and IFN-β as the most potent SARS-CoV-2 replication inhibitors among this class of molecules, also acting on Delta and Omicron strains – even if at a lesser extent compared to ancestral isolates (58).

Data from COVID-19 patients underline the relevance of an early, efficient IFN response to block disease progression. Patients with severe COVID-19 display low induction of systemic and local (i.e., in the airway epithelium) IFN secretion (59) in the early phases, which traces back to a hypo responsiveness of IFN-producing plasmacytoid dendritic cells to viral infection (60). Conversely, children and people with mild disease show strong IFN-mediated innate activation in the upper airways, driven by MDA5 and RIG-I impeding viral dissemination to the lungs (61–64).

Age is a risk factor for COVID-19-related death. Perhaps, the immune-senescence processes also involve poorer and delayed IFN-mediated innate responses, plausibly due to an impairment in RIG-I signaling (65, 66). Comparing adult and aged COVID-19 mouse models, Beer and colleagues demonstrated that an age-dependent increase in disease severity depends on an impaired IFN I-II response which caused a higher viral load and a delayed, uncoordinated adaptive immunity (in terms of antigen presentation, NK lymphocytes activation, and immunoglobulin production) (67).

Broadening this concept, “IFN deficiency” is associated with a worse prognosis from COVID-19 at all ages. Indeed, several inborn defects of IFN immunity have been found in critical (especially younger) COVID-19 patients: autosomal recessive deficiencies of *IFNAR1* and *IRF7* (1.8% of cases), *X-linked* deficiency of *TLR7 (1.3%)*, newly described or known autosomal dominant deficiencies of *IFNAR1, IFNAR2, TLR3, IRF3, TRF, TBK1, UNC93B1* (2.9%), enhancing the risk of a severe disease course 20 to 34-fold (68–70). Recessive deficiencies of type I IFN immunity may underlie ∼10% of pediatric COVID-19 hospitalizations (70). Smaller effects (odds ratios ~1.5 to 2.1) are attributed to common variants identified by COVID-19 GWAS studies, linked to genes involved in IFN signaling: risk SNPs in *TYK2* and *IFNAR2* genes, protective polymorphisms in the OAS1, OAS2, OAS3 cluster of genes, which are ISGs (71–74). Other variants impacting IFN induction, such as those of the RNA sensor MDA5, exert their effects on disease severity in an age-dependent fashion, plausibly due to the failure of compensatory immunological synergies in the elderly (75, 76)

Acquired autoimmune phenocopies of IFN deficiencies may account for ~20% of COVID-19-related deaths. At first, Bastard and colleagues identified autoantibodies against IFN-α or IFN-ω in ~13.6% of patients with critical COVID-19, with age-related prevalence (21% in patients >80 years), while anti-IFN-β antibodies were found in ~1.3% severe COVID-19 patients, independently from age. Considering a significantly lower prevalence in the general population (0.18-3.4%), the presence of anti-IFN antibodies appeared to increase the susceptibility towards a severe SARS-CoV-2 infection (77). The result of anti-IFN α/ω was replicated in independent cohorts, allowing to mechanistically link the presence of auto-antibodies to a dampened IFN signature at the transcriptomic level as well as to lymphopenia, higher systemic inflammation indexes and poorer outcomes (78–80). The presence and role of anti-IFN-β antibodies did not receive such a strong confirmation (81), supporting the possibility of a fruitful usage of IFN-β as a therapeutic solution in the course of COVID-19 (82) (**Figure 1**).

Since the first months of the pandemic, several trials have tested IFN-β formulations (either IFN-β1a or IFN-β1b) in COVID-19, whose results have been reviewed and meta-analyzed recently (83–87). Overall, the data indicate that IFN-β administration could decrease symptoms severity, ICU admission, and - in some studies - overall hospital stay; however, effects on mortality reduction did not reach statistical significance in recent pooled analysis (83, 84).

To interpret such data some caveats should be considered. There was substantial heterogeneity in enrollment criteria and in co-administered drugs: for example, the contemporary usage of corticosteroids could hamper IFN-β immune-modulating efficacy (88). Most importantly, the timing of IFN-β treatment initiation could be crucial: while a benefit is expected in the early phase of the infection to halt virus spread and enhance immunity, delayed dosing could perhaps foster immunopathology and cytokine overproduction (89, 90). Indeed, in most of the trials, the benefit on symptoms and mortality emerged more clearly in patients receiving IFN-β in the first days of symptomatic disease (86); contrariwise, in a recently published RCT showing no superiority of Interferon β-1a plus remdesivir to remdesivir alone in hospitalized patients with COVID-19 pneumonia, the mean duration of symptoms before enrolment was 8.7 days in the IFN-β/remdesivir arm (91). In conclusion, the use of IFN-β would appear more appropriate in recently infected subjects, ideally screened for IFN signature, with direct (such as plasma IFN-B level) or surrogate biomarkers (such as neutrophil/lymphocyte ratio) (92–94).

## Conclusions and future directions

While IFN-β in MS is turning 30, the last three years taught about its timeliness, for both pwMS and the general population. The renewed interest in IFN biology related to viral infections (and vaccinations) has led to a deeper knowledge on the effects of these molecules on the immune system, which could be rapidly translated into practice thanks to solid experience in the MS field.

In the last decades, there has been a striking expansion of MS therapeutic armamentarium, improving the prognosis and quality of life of pwMS. Currently approved DMTs can be classified based on their efficacy (in reducing relapses and disability accumulation) in first-line/moderate efficacy drugs, such as IFNs, glatiramer acetate, teriflunomide and dymethil fumarate) and second-line/high efficacy therapies (HET) (S1PR modulators, cladribine, natalizumab, anti-CD20 monoclonal antibodies and alemtuzumab). While traditionally first-line agents were chosen at disease onset and patients were shift to higher efficacy drugs in case of treatment failure or suboptimal response (“escalation” strategy), there is increasing evidence supporting the early use of HET (“induction” strategy) to guarantee a better control of disability accumulation in the long term (95–97). However, the increasing efficacy in preventing disease activity comes along with increased risks of adverse events, such as infections and, rarely, neoplasms, related to broad immunosuppressive effects of HET. Beyond safety and tolerability and the risk-benefit profile, the choice of DMT should be tailored on each patient accounting for disease phenotype, comorbidities, patient’s age, personal preferences, reproductive planning, costs, and access (98). In such a scenario, IFN-β shows an excellent safety profile paired with moderate efficacy; also, it is approved for pregnancy and during breastfeeding, allowing its use in women with MS who are planning a pregnancy (99). The low infectious risks - and the suggested effect against viral infections (100–103), as for COVID-19 - also make it suitable for elderly pwMS, such as in late-onset MS. Indeed, as the average age of onset of MS increases (104), a greater number of elderly pwMS is facing the need for DMTs that account for higher risks of neoplasms and infections in this subpopulation (105). As discussed for SARS-CoV-2, impaired type I IFN production and response is part of immunosenescence and contributes to the increased susceptibility to infections, not only due to the lack of a prompt innate antimicrobial activation but also because of weakened T and B adaptive responses to vaccinations (106). IFN dysregulation in the elderly is not simply quantitative, as it involves a wider unbalance between “tonic” and “phasic” IFN secretion that can lead to a paradoxical chronic, low-grade inflammatory state fueling tissue aging (107). Intriguingly, a lower type I IFN activity was also linked to an accelerated cognitive decline toward overt dementia syndrome (108). Therefore, IFN-β could represents a *smart* therapeutic option for elderly pwMS, combining an optimal safety profile with broad immunomodulating effects that go beyond neuroinflammation. New and more convenient therapeutic schedules may also be explored (109).

Efficient vaccines and direct antivirals have been developed to prevent and treat SARS-CoV-2 infection, quenching the urge for IFN-β repurposing in COVID-19. However, a future challenge will be the management of the multisystemic post-acute COVID-19 symptoms (PASC, or long COVID-19) (110), whose prevalence is estimated to exceed 40% of infected people (111). Its pathophysiology is not fully understood, but persistent dysimmunity links this condition to other post-viral diseases, including the chronic fatigue syndrome that follows infectious mononucleosis (112). It is of interest that EBV reactivation in the acute phase emerged as a prominent risk factor for long-COVID-19 (113, 114). Future studies evaluating whether IFN-β protects pwMS also from long-COVID-19 could shed light on a possible role of this molecule in this setting. Also, it will be crucial to evaluate the interplay between SARS-CoV-2 and EBV in-depth and aside from COVID-19, as potential viral synergies could have implications for MS’s natural history (115).

## Author contributions

GB: conceptualization, data curation, formal analysis, investigation, methodology, writing – original draft, writing – review and editing. AA: Conceptualization, formal analysis, investigation, methodology, writing – original draft, writing – review and editing. CR: Conceptualization, formal analysis, investigation, methodology, writing – original draft, writing – review and editing. VR: Conceptualization, writing – review and editing. MS: Conceptualization, methodology, supervision, validation, writing – review and editing. GR: Conceptualization, methodology, supervision, validation, writing – review and editing. All authors contributed to the article and approved the submitted version.

## References

[1] Charabati M Wheeler MA Weiner HL Quintana FJ. Multiple sclerosis: neuroimmune crosstalk and therapeutic targeting. Cell (2023) 186:1309–27. doi: 10.1016/j.cell.2023.03.008 PMC10119687 37001498

[2] Bjornevik K Cortese M Healy BC Kuhle J Mina MJ Leng Y. Longitudinal analysis reveals high prevalence of Epstein-Barr virus associated with multiple sclerosis. Science (2022) 375:296–301. doi: 10.1126/SCIENCE.ABJ8222/SUPPL_FILE/SCIENCE.ABJ8222_MDAR_REPRODUCIBILITY_CHECKLIST.PDF 35025605

[3] Lanz TV Brewer RC Ho PP Moon JS Jude KM Fernandez D. Clonally expanded b cells in multiple sclerosis bind EBV EBNA1 and GlialCAM. Nature (2022) 603:321–7. doi: 10.1038/s41586-022-04432-7 PMC9382663 35073561

[4] Aloisi F Giovannoni G Salvetti M. Epstein-Barr Virus as a cause of multiple sclerosis: opportunities for prevention and therapy. Lancet Neurol (2023) 22:338–49. doi: 10.1016/S1474-4422(22)00471-9 36764322

[5] Pugliatti M Berger T Hartung HP Oreja-Guevara C Bar-Or A. Multiple sclerosis in the era of COVID-19: disease course, DMTs and SARS-CoV2 vaccinations. Curr Opin Neurol (2022) 35:319–27. doi: 10.1097/WCO.0000000000001066 35674075

[6] Reder AT Centonze D Naylor ML Nagpal A Rajbhandari R Altincatal A. COVID-19 in patients with multiple sclerosis: associations with disease-modifying therapies. CNS Drugs (2021) 35:317–30. doi: 10.1007/S40263-021-00804-1 PMC7980129 33743151

[7] Simpson-Yap S De Brouwer E Kalincik T Rijke N Hillert JA Walton C. Associations of disease-modifying therapies with COVID-19 severity in multiple sclerosis. Neurology (2021) 97:E1870–85. doi: 10.1212/WNL.0000000000012753 PMC8601210 34610987

[8] Sormani MP De Rossi N Schiavetti I Carmisciano L Cordioli C Moiola L. Disease-modifying therapies and coronavirus disease 2019 severity in multiple sclerosis. Ann Neurol (2021) 89:780–9. doi: 10.1002/ana.26028 PMC8013440 33480077

[9] Sormani MP Schiavetti I Carmisciano L Cordioli C Filippi M Radaelli M. COVID-19 severity in multiple sclerosis: putting data into context. Neurology(R) Neuroimmunol Neuroinflamm (2022) 9(1):e1105. doi: 10.1212/NXI.0000000000001105 PMC8579249 34753829

[10] Iaffaldano P Lucisano G Manni A Paolicelli D Patti F Capobianco M. Risk of getting COVID-19 in people with multiple sclerosis. Neurol - Neuroimmunol Neuroinflamm (2022) 9:1141. doi: 10.1212/NXI.0000000000001141 PMC8771668 35046084

[11] Sormani MP Salvetti M Labauge P Schiavetti I Zephir H Carmisciano L. DMTs and covid-19 severity in MS: a pooled analysis from Italy and France. Ann Clin Trans Neurol (2021) 8(8):1738–44. doi: 10.1002/ACN3.51408 PMC8351392 34240579

[12] Jakimovski D Kolb C Ramanathan M Zivadinov R Weinstock-Guttman B. Interferon β for multiple sclerosis. Cold Spring Harbor Perspect Med (2018) 9(1):e1105. doi: 10.1101/CSHPERSPECT.A032003 PMC6211378 29311124

[13] McNab F Mayer-Barber K Sher A Wack A O'Garra A. Type I interferons in infectious disease. Nat Rev Immunol (2015) 15:87–103. doi: 10.1038/nri3787 25614319 PMC7162685

[14] Isaacs A Lindemann J. Virus interference. i. the interferon. Proc R Soc Lond Ser B Biol Sci (1957) 147:258–67. 26297790

[15] Schneider WM Chevillotte MD Rice CM. Interferon-stimulated genes: a complex web of host defenses. Annu Rev Immunol (2014) 32:513–45. doi: 10.1146/annurev-immunol-032713-120231 PMC4313732 24555472

[16] Rizzo F Giacomini E Mechelli R. Interferon-β therapy specifically reduces pathogenic memory b cells in multiple sclerosis patients by inducing a FAS-mediated apoptosis. Immunol Cell Biol (2016) 94:886–94. doi: 10.1038/icb.2016.55 27265253

[17] Annibali V Umeton R Palermo A Severa M Paola M Giglio S. Analysis of coding and non-coding transcriptome of peripheral b cells reveals an altered interferon response factor (IRF) -1 pathway in multiple sclerosis patients. J Neuroimmunol (2018) 324:165–71. doi: 10.1016/j.jneuroim.2018.09.005 30270021

[18] Feng X Petraglia AL Chen M Byskosh PV Boos MD Reder AT. Low expression of interferon-stimulated genes in active multiple sclerosis is linked to subnormal phosphorylation of STAT1. J Neuroimmunol (2002) 129:205–15. doi: 10.1016/S0165-5728(02)00182-0 12161037

[19] Severa M Rizzo F Srinivasan S Di Dario M Giacomini E Buscarinu MC. A cell type-specific transcriptomic approach to map b cell and monocyte type I interferon-linked pathogenic signatures in multiple sclerosis. J Autoimmun (2019) 101:1–16. doi: 10.1016/j.jaut.2019.04.006 31047767

[20] Srinivasan S Severa M Rizzo F Menon R Brini E Mechelli R. Transcriptional dysregulation of interferome in experimental and human multiple sclerosis. Sci Rep (2017) 7:8981. doi: 10.1038/s41598-017-09286-y 28827704 PMC5566335

[21] Beecham AH Patsopoulos NA Xifara DK Davis MF Kemppinen A Cotsapas C. Analysis of immune-related loci identifies 48 new susceptibility variants for multiple sclerosis. Nat Genet (2013) 45:1353–60. doi: 10.1038/ng.2770 PMC3832895 24076602

[22] Patsopoulos NA Baranzini SE Santaniello A Shoostari P Cotsapas C Wong G. Multiple sclerosis genomic map implicates peripheral immune cells and microglia in susceptibility. Sci (New York NY) (2019) 365. doi: 10.1126/SCIENCE.AAV7188 PMC7241648 31604244

[23] Mechelli R Romano C Reniè R Manfrè G Buscarinu MC Romano S. Viruses and neuroinflammation in multiple sclerosis. Neuroimmunol Neuroinflamm (2021) 8:269. doi: 10.20517/2347-8659.2021.01

[24] Damania B Kenney SC Raab-Traub N. Epstein-Barr Virus: biology and clinical disease. Cell (2022) 185(20):3652–70. doi: 10.1016/j.cell.2022.08.026 PMC9529843 36113467

[25] Jog NR McClain MT Heinlen LD Gross T Towner R Guthridge JM. Epstein Barr Virus nuclear antigen 1 (EBNA-1) peptides recognized by adult multiple sclerosis patient sera induce neurologic symptoms in a murine model. J Autoimmun (2019) 1:102332. doi: 10.1016/j.jaut.2019.102332 PMC6930324 31515129

[26] Tengvall K Huang J Hellström C Kammer P Biström M Ayoglu B. Molecular mimicry between anoctamin 2 and Epstein-Barr virus nuclear antigen 1 associates with multiple sclerosis risk. Proc Natl Acad Sci United States America (2019) 116:16955–60. doi: 10.1073/PNAS.1902623116 PMC6708327 31375628

[27] D’Addario M Libermann TA Xu J Ahmad A Menezes J. Epstein-Barr Virus and its glycoprotein-350 upregulate IL-6 in human b-lymphocytes *via* CD21, involving activation of NF-κB and different signaling pathways. J Mol Biol (2001) 308(3):501–14. doi: 10.1006/jmbi.2001.4589 11327783

[28] Kang MS Kieff E. Epstein-Barr Virus latent genes. Exp Mol Med (2015) 47:e131. doi: 10.1038/emm.2014.84 25613728 PMC4314583

[29] Serafini B Rosicarelli B Franciotta D Magliozzi R Reynolds R Cinque P. Dysregulated Epstein-Barr virus infection in the multiple sclerosis brain. J Exp Med (2007) 204(12):2899–912. doi: 10.1084/jem.20071030 PMC2118531 17984305

[30] Bar-Or A Pender MP Khanna R Steinman L Hartung HP Maniar T. Epstein–Barr Virus in multiple sclerosis: theory and emerging immunotherapies. Trends Mol Med (2019) 8(23):296–310. doi: 10.1016/j.molmed.2019.11.003 PMC7106557 31862243

[31] Mechelli R Umeton R Policano C Annibali V Coarelli G Ricigliano VAG. A “Candidate-interactome” aggregate analysis of genome-wide association data in multiple sclerosis. PloS One (2013) 8(5):e63300. doi: 10.1371/journal.pone.0063300 23696811 PMC3655974

[32] Mechelli R Manzari C Policano C Annese A Picardi E Umeton R. Epstein-Barr Virus genetic variants are associated with multiple sclerosis. Neurology (2015) 84(13):1362–8. doi: 10.1212/WNL.0000000000001420 PMC4388746 25740864

[33] Harley JB Chen X Pujato M Miller D Maddox A Forney C. Transcription factors operate across disease loci, with EBNA2 implicated in autoimmunity. Nat Genet (2018) 50:699–707. doi: 10.1038/s41588-018-0102-3 PMC6022759 29662164

[34] Fierz W. Multiple sclerosis: an example of pathogenic viral interaction? Virol J (2017) 14:42. doi: 10.1186/s12985-017-0719-3 28241767 PMC5330019

[35] Küry P Nath A Créange A Dolei A Marche P Gold J. Human endogenous retroviruses in neurological diseases. Trends Mol Med (2018) 24(4):379–94. doi: 10.1016/j.molmed.2018.02.007 PMC7185488 29551251

[36] Höllsberg P Kusk M Bech E Hansen HJ Jakobsen J Haahr S. Presence of Epstein-Barr virus and human herpesvirus 6B DNA in multiple sclerosis patients: associations with disease activity. Acta Neurol Scand (2005) 112:395–402. doi: 10.1111/j.1600-0404.2005.00516.x 16281923

[37] Torkildsen Nyland H Myrmel H Myhr KM. Epstein-Barr Virus reactivation and multiple sclerosis. Eur J Neurol (2008) 15:106–8. doi: 10.1111/j.1468-1331.2007.02009.x 18042233

[38] Wandinger KP Jabs W Siekhaus A Bubel S Trillenberg P Wagner HJ. Association between clinical disease activity and Epstein-Barr virus reactivation in MS. Neurology (2000) 55:178–84. doi: 10.1212/WNL.55.2.178 10908887

[39] Lange PT White MC Damania B. Activation and evasion of innate immunity by gammaherpesviruses. J Mol Biol (2022) 434:167214. doi: 10.1016/J.JMB.2021.167214 34437888 PMC8863980

[40] Bentz GL Liu R Hahn AM Shackelford J Pagano JS. Epstein-Barr Virus BRLF1 inhibits transcription of IRF3 and IRF7 and suppresses induction of interferon-β. Virology (2010) 402:121–8. doi: 10.1016/J.VIROL.2010.03.014 PMC2871977 20381110

[41] Liu X Sadaoka T Krogmann T Cohen JI. Epstein-Barr Virus (EBV) tegument protein BGLF2 suppresses type I interferon signaling to promote EBV reactivation. J Virol (2020) 94. doi: 10.1128/JVI.00258-20 PMC7269453 32213613

[42] Wang J-T Doong S-L Teng S-C Lee C-P Tsai C-H Chen M-R. Epstein-Barr Virus BGLF4 kinase suppresses the interferon regulatory factor 3 signaling pathway. J Virol (2009) 83:1856–69. doi: 10.1128/JVI.01099-08 PMC2643756 19052084

[43] Wu L Fossum E Joo CH Inn K-S Shin YC Johannsen E. Epstein-Barr Virus LF2: an antagonist to type I interferon. J Virol (2009) 83:1140–6. doi: 10.1128/JVI.00602-08 PMC2612359 18987133

[44] Giacomini E Severa M Rizzo F Mechelli R Annibali V Ristori G. IFN-β therapy modulates b-cell and monocyte crosstalk *via* TLR7 in multiple sclerosis patients. Eur J Immunol (2013) 43:1963–72. doi: 10.1002/eji.201243212 23636665

[45] Hislop AD Taylor GS Sauce D Rickinson AB. Cellular responses to viral infection in humans: lessons from Epstein-Barr virus. Annu Rev Immunol (2007) 25:587–617. doi: 10.1146/annurev.immunol.25.022106.141553 17378764

[46] Recher M Lang KS Navarini A Hunziker L Lang PA Fink K. Extralymphatic virus sanctuaries as a consequence of potent T-cell activation. Nat Med (2007) 13:1316–23. doi: 10.1038/nm1670 PMC7096094 17982463

[47] Angelini DF Serafini B Piras E Severa M Coccia EM Rosicarelli B. Increased CD8+ T cell response to Epstein-Barr virus lytic antigens in the active phase of multiple sclerosis. PloS Pathog (2013) 9(4):e1003220. doi: 10.1371/journal.ppat.1003220 23592979 PMC3623710

[48] Comabella M Kakalacheva K Río J Münz C Montalban X Lünemann JD. EBV-specific immune responses in patients with multiple sclerosis responding to IFNβ therapy. Multiple Sclerosis J (2012) 18:605–9. doi: 10.1177/1352458511426816 22020417

[49] Hu B Guo H Zhou P. Characteristics of SARS-CoV-2 and COVID-19. Nat Rev Microbiol (2021) 19:141–54. doi: 10.1038/s41579-020-00459-7 PMC7537588 33024307

[50] Lucas C Wong P Klein J Castro TBR Silva J Sundaram M. Longitudinal analyses reveal immunological misfiring in severe COVID-19. Nat 2020 584:7821 (2020) 584:463–9. doi: 10.1038/s41586-020-2588-y PMC7477538 32717743

[51] Lamers MM Haagmans BL. SARS-CoV-2 pathogenesis. Nat Rev Microbiol 2022 20:5 (2022) 20:270–84. doi: 10.1038/s41579-022-00713-0 35354968

[52] Jouvenet N Goujon C Banerjee A. Clash of the titans: interferons and SARS-CoV-2. Trends Immunol (2021) 42:1069–72. doi: 10.1016/j.it.2021.10.009 PMC8519778 34742657

[53] Lei X Dong X Ma R Wang W Xiao X Tian Z. Activation and evasion of type I interferon responses by SARS-CoV-2. Nat Commun (2020) 11:3810. doi: 10.1038/S41467-020-17665-9 32733001 PMC7392898

[54] Minkoff JM tenOever B. Innate immune evasion strategies of SARS-CoV-2. Nat Rev Microbiol (2023) 21:178–94. doi: 10.1038/s41579-022-00839-1 PMC9838430 36631691

[55] Xia H Cao Z Xie X Zhang X Chen JYC Wang H. Evasion of type I interferon by SARS-CoV-2. Cell Rep (2020) 33:108234. doi: 10.1016/j.celrep.2020.108234 32979938 PMC7501843

[56] Sui L Zhao Y Wang W Wu P Wang Z Yu Y. SARS-CoV-2 membrane protein inhibits type I interferon production through ubiquitin-mediated degradation of TBK1. Front Immunol (2021) 12:662989. doi: 10.3389/FIMMU.2021.662989 34084167 PMC8168463

[57] Lokugamage KG Hage A de Vries M Valero-Jimenez AM Schindewolf C Dittmann M. Type I interferon susceptibility distinguishes SARS-CoV-2 from SARS-CoV. J Virol (2020) 94(23):e01410–20. doi: 10.1128/JVI.01410-20 PMC7654262 32938761

[58] Guo K Barrett BS Morrison JH Mickens KL Vladar EK Hasenkrug KJ. Interferon resistance of emerging SARS-CoV-2 variants. Proc Natl Acad Sci United States America (2022) 119:e2203760119. doi: 10.1073/PNAS.2203760119/SUPPL_FILE/PNAS.2203760119.SAPP.PDF PMC9371743 35867811

[59] Park A Iwasaki A. Type I and type III interferons – induction, signaling, evasion, and application to combat COVID-19. Cell Host Microbe (2020) 27:870–8. doi: 10.1016/J.CHOM.2020.05.008 PMC7255347 32464097

[60] Venet M Ribeiro MS Décembre E Bellomo A Joshi G Nuovo C. Severe COVID-19 patients have impaired plasmacytoid dendritic cell-mediated control of SARS-CoV-2. Nat Commun (2023) 14:694. doi: 10.1038/s41467-023-36140-9 36755036 PMC9907212

[61] Hadjadj J Yatim N Barnabei L Corneau A Boussier J Smith N. Impaired type I interferon activity and inflammatory responses in severe COVID-19 patients. Science (2020) 369:718–24. doi: 10.1126/science.abc6027 PMC7402632 32661059

[62] Loske J Röhmel J Lukassen S Stricker S Magalhães VG Liebig J. Pre-activated antiviral innate immunity in the upper airways controls early SARS-CoV-2 infection in children. Nat Biotechnol (2022) 40:319–24. doi: 10.1038/S41587-021-01037-9 34408314

[63] Sungnak W Huang N Bécavin C Berg M Queen R Litvinukova M. SARS-CoV-2 entry factors are highly expressed in nasal epithelial cells together with innate immune genes. Nat Med (2020) 26:681–7. doi: 10.1038/s41591-020-0868-6 PMC8637938 32327758

[64] Yoshida M Worlock KB Huang N. Local and systemic responses to SARS-CoV-2 infection in children and adults. Nature (2022) 602:321–7. doi: 10.1038/s41586-021-04345-x PMC8828466 34937051

[65] Bartleson JM Radenkovic D Covarrubias AJ Furman D Winer DA Verdin E. SARS-CoV-2, COVID-19 and the aging immune system. Nat Aging 2021 1:9 (2021) 1:769–82. doi: 10.1038/s43587-021-00114-7 PMC8570568 34746804

[66] Deng J Zheng S-N Xiao Y Nan M-L Zhang J Han L. SARS-CoV-2 NSP8 suppresses type I and III IFN responses by modulating the RIG-I/MDA5, TRIF, and STING signaling pathways. J Med Virol (2023) 95(4). doi: 10.1002/jmv.28680 36929724

[67] Beer J Crotta S Breithaupt A Ohnemus A Becker J Sachs B. Impaired immune response drives age-dependent severity of COVID-19. J Exp Med (2022) 219(12):e20220621. doi: 10.1084/JEM.20220621 36129445 PMC9499827

[68] Zhang Q Bastard P Karbuz A Gervais A Tayoun AA Aiuti A. Human genetic and immunological determinants of critical COVID-19 pneumonia. Nat 2022 603:7902 (2022) 603:587–98. doi: 10.1038/s41586-022-04447-0 PMC8957595 35090163

[69] Zhang Q Bastard P Liu Z. Inborn errors of type I IFN immunity in patients with life-threatening COVID-19. Science (2020) 370:eabd4570. doi: 10.1126/science.abd4570. 2020 09 26. 32972995 PMC7857407

[70] Zhang Q Matuozzo D Le Pen J Lee D Moens L Asano T. Recessive inborn errors of type I IFN immunity in children with COVID-19 pneumonia. J Exp Med (2022) 219. doi: 10.1084/JEM.20220131/213287 PMC9206114 35708626

[71] Asano T Boisson B Onodi F Matuozzo D Moncada-Velez M Renkilaraj MRLM. X-Linked recessive TLR7 deficiency in ~1% of men under 60 years old with life-threatening COVID-19. Sci Immunol (2021) 6:eabl4348. doi: 10.1126/sciimmunol.abl4348 34413140 PMC8532080

[72] Niemi MEK Karjalainen J Liao RG Neale BM Daly M Ganna A. Mapping the human genetic architecture of COVID-19. Nature (2021) 600:472–7. doi: 10.1038/s41586-021-03767-x PMC8674144 34237774

[73] Pairo-Castineira E Clohisey S Klaric L Bretherick AD Rawlik K Pasko D. Genetic mechanisms of critical illness in COVID-19. Nature (2021) 591:92–8. doi: 10.1038/s41586-020-03065-y 33307546

[74] Matuozzo D Talouarn E Marchal A Zhang P Manry J Seeleuthner Y. Rare predicted loss-of-function variants of type I IFN immunity genes are associated with life-threatening COVID-19. Genome Med (2023) 15:22. doi: 10.1186/s13073-023-01173-8 37020259 PMC10074346

[75] Muñiz-Banciella MG Albaiceta GM Amado-Rodríguez L Del Riego ES Alonso IL López-Martínez C. Age-dependent effect of the IFIH1/MDA5 gene variants on the risk of critical COVID-19. Immunogenetics (2023) 75:91–8. doi: 10.1007/s00251-022-01281-6 PMC9702716 36434151

[76] Amado-Rodríguez L Salgado Del Riego E Gomez de Ona J López Alonso I Gil-Pena H López-Martínez C. Effects of IFIH1 rs1990760 variants on systemic inflammation and outcome in critically ill COVID-19 patients in an observational translational study. Elife (2022) 11:e73012. doi: 10.7554/eLife.73012 35060899 PMC8782569

[77] Bastard P Gervais A Voyer TL Rosain J Philippot Q Manry J. Autoantibodies neutralizing type I IFNs are present in ~4% of uninfected individuals over 70 years old and account for ~20% of COVID-19 deaths. Sci Immunol (2021) 6:eabl4340. doi: 10.1126/sciimmunol.abl4340 34413139 PMC8521484

[78] Frasca F Scordio M Santinelli L Gabriele L Gandini O Criniti A. Anti-IFN-α/-ω neutralizing antibodies from COVID-19 patients correlate with downregulation of IFN response and laboratory biomarkers of disease severity. Eur J Immunol (2022) 52:1120–8. doi: 10.1002/EJI.202249824 PMC9087404 35419822

[79] Goncalves D Mezidi M Bastard P Perret M Saker K Fabien N. Antibodies against type I interferon: detection and association with severe clinical outcome in COVID-19 patients. Clin Trans Immunol (2021) 10(8):e1327. doi: 10.1002/CTI2.1327 PMC8370568 34429968

[80] van der Wijst MGP Vazquez SE Hartoularos GC Bastard P Grant T Bueno R. Type I interferon autoantibodies are associated with systemic immune alterations in patients with COVID-19. Sci Transl Med (2021) 13:eabh2624. doi: 10.1126/SCITRANSLMED.ABH2624 34429372 PMC8601717

[81] Troya J Bastard P Planas-Serra L Ryan P Ruiz M de Carranza M. Neutralizing autoantibodies to type I IFNs in >10% of patients with severe COVID-19 pneumonia hospitalized in Madrid, Spain. J Clin Immunol (2021) 41:914–22. doi: 10.1007/S10875-021-01036-0 PMC8043439 33851338

[82] Hale BG. Autoantibodies targeting type I interferons: prevalence, mechanisms of induction, and association with viral disease susceptibility. Eur J Immunol (2023), e2250164. doi: 10.1002/eji.202250164 37027328

[83] Crichton ML Goeminne PC Tuand K Vandendriessche T Tonia T Roche N. The impact of therapeutics on mortality in hospitalised patients with COVID-19: systematic review and meta-analyses informing the European respiratory society living guideline. Eur Respir Rev (2021) 30. doi: 10.1183/16000617.0171-2021 PMC8796659 34911695

[84] Kumar S Saurabh MK Narasimha VL Maharshi V. Efficacy of interferon-β in moderate-to-Severe hospitalised cases of COVID-19: a systematic review and meta-analysis. Clin Drug Invest (2021) 41:1037–46. doi: 10.1007/S40261-021-01092-9/TABLES/2 PMC8540871 34687413

[85] Nakhlband A Fakhari A Azizi H. Interferon-beta offers promising avenues to COVID-19 treatment: a systematic review and meta-analysis of clinical trial studies. Naunyn-Schmiedeberg’s Arch Pharmacol (2021) 394:829–38. doi: 10.1007/S00210-021-02061-X PMC7883756 33587164

[86] Sosa JP Caceres MMF Comptis JR Quiros J Príncipe-Meneses FS Riva-Moscoso A. Effects of interferon beta in covid-19 adult patients: systematic review. Infection Chemother (2021) 53:247–60. doi: 10.3947/IC.2021.0028 PMC8258298 34216119

[87] Ryoo S Koh D-H Yu S-Y Choi M Huh K Yeom J-S. Clinical efficacy and safety of interferon (Type I and type III) therapy in patients with COVID-19: a systematic review and meta-analysis of randomized controlled trials. PloS One (2023) 18:e0272826. doi: 10.1371/journal.pone.0272826 36989209 PMC10057835

[88] Jalkanen J Pettilä V Huttunen T Hollmén M Jalkanen S. Glucocorticoids inhibit type I IFN beta signaling and the upregulation of CD73 in human lung. Intensive Care Med (2020) 46:1937–40. doi: 10.1007/S00134-020-06086-3 PMC7235433 32430515

[89] Akamatsu MA de Castro JT Takano CY Ho PL. Off balance: interferons in COVID-19 lung infections. EBioMedicine (2021) 73. doi: 10.1016/J.EBIOM.2021.103642 PMC8524139 34678609

[90] Galani IE Rovina N Lampropoulou V Triantafyllia V Manioudaki M Pavlos E. Untuned antiviral immunity in COVID-19 revealed by temporal type I/III interferon patterns and flu comparison. Nat Immunol (2021) 22:32–40. doi: 10.1038/S41590-020-00840-X 33277638

[91] Kalil AC Mehta AK Patterson TF Erdmann N Gomez CA Jain MK. Efficacy of interferon beta-1a plus remdesivir compared with remdesivir alone in hospitalised adults with COVID-19: a double-bind, randomised, placebo-controlled, phase 3 trial. Lancet Respir Med (2021) 9:1365–76. doi: 10.1016/S2213-2600(21)00384-2 PMC8523116 34672949

[92] Galbraith MD Kinning KT Sullivan KD Araya P Smith KP Granrath RE. Specialized interferon action in COVID-19. Proc Natl Acad Sci United States America (2022) 119(11):e2116730119. doi: 10.1073/PNAS.2116730119 PMC8931386 35217532

[93] Darif D Ejghal R Desterke C Outlioua A Hammi I Lemrani M. Type I and III interferons are good markers to monitor COVID-19 pathophysiology. Cytokine (2023) 165:156172. doi: 10.1016/j.cyto.2023.156172 36924609 PMC10008794

[94] Berri F N’Guyen Y Callon D Lebreil A-L Glenet M Heng L. Early plasma interferon-β levels as a predictive marker of COVID-19 severe clinical events in adult patients. J Med Virol (2023) 95:e28361. doi: 10.1002/jmv.28361 36451263 PMC9877952

[95] Harding K Williams O Willis M Hrastelj J Rimmer A Joseph F. Clinical outcomes of escalation vs early intensive disease-modifying therapy in patients with multiple sclerosis. JAMA Neurol (2019) 76(5):536–41. doi: 10.1001/jamaneurol.2018.4905 PMC6515582 30776055

[96] Prosperini L Mancinelli CR Solaro CM Nociti V Haggiag S Cordioli C. Induction versus escalation in multiple sclerosis: a 10-year real world study. Neurotherapeutics (2020) 17:994–1004. doi: 10.1007/s13311-020-00847-0 32236822 PMC7609676

[97] Casanova B Quintanilla-Bordás C Gascón F. Escalation vs. early intense therapy in multiple sclerosis. J Pers Med (2022) 12:119. doi: 10.3390/jpm12010119 35055434 PMC8778390

[98] Berger T Adamczyk-Sowa M Csépány T Fazekas F Fabjan TH Horáková D. Factors influencing daily treatment choices in multiple sclerosis: practice guidelines, biomarkers and burden of disease. Ther Adv Neurol Disord (2020) 13:1756286420975223. doi: 10.1177/1756286420975223 33335562 PMC7724259

[99] Varytė G Arlauskienė A Ramašauskaitė D. Pregnancy and multiple sclerosis: an update. Curr Opin Obstet Gynecol (2021) 33:378–83. doi: 10.1097/GCO.0000000000000731 PMC8452312 34310364

[100] Evans C Zhu F Kingwell E Shirani A van der Kop ML Petkau J. Association between beta-interferon exposure and hospital events in multiple sclerosis. Pharmacoepidemiol Drug Saf (2014) 23:1213–22. doi: 10.1002/pds.3667 24953054

[101] Dhib-Jalbut S Marks S. Interferon-β mechanisms of action in multiple sclerosis. Neurology (2010) 74. doi: 10.1212/WNL.0B013E3181C97D99 20038758

[102] de Jong HJI Kingwell E Shirani A Cohen Tervaert JW Hupperts R Zhao Y. Evaluating the safety of β-interferons in MS: a series of nested case-control studies. Neurology (2017) 88:2310–20. doi: 10.1212/WNL.0000000000004037 PMC5567323 28500224

[103] Oikonen MK Erälinna JP. Beta-interferon protects multiple sclerosis patients against enhanced susceptibility to infections caused by poor air quality. Neuroepidemiology (2008) 30:13–9. doi: 10.1159/000113301 18204292

[104] Prosperini L Lucchini M Ruggieri S Tortorella C Haggiag S Mirabella M. Shift of multiple sclerosis onset towards older age. J Neurol Neurosurg Psychiatry (2022) 93:1137–9. doi: 10.1136/JNNP-2022-329049 35477891

[105] Buscarinu MC Reniè R Morena E Romano C Bellucci G Marrone A. Late-onset MS: disease course and safety-efficacy of DMTS. Front Neurol (2022) 13:829331/BIBTEX. doi: 10.3389/FNEUR.2022.829331/BIBTEX 35356454 PMC8960027

[106] Feng E Balint E Poznanski SM Ashkar AA Loeb M. Aging and interferons: impacts on inflammation and viral disease outcomes. Cells (2021) 10(3):708. doi: 10.3390/CELLS10030708 33806810 PMC8004738

[107] Cao W. IFN-aging: coupling aging with interferon response. Front Aging (2022) 0:870489. doi: 10.3389/FRAGI.2022.870489 PMC9261325 35821859

[108] Song L Chen J Lo CYZ Guo Q Feng J Zhao XM. Impaired type I interferon signaling activity implicated in the peripheral blood transcriptome of preclinical alzheimer’s disease. eBioMedicine (2022) 82. doi: 10.1016/j.ebiom.2022.104175 PMC9304603 35863293

[109] Romano S Ferraldeschi M Bagnato F Mechelli R Morena E Caldano M. Drug holiday of interferon beta 1b in multiple sclerosis: a pilot, randomized, single blind study of non-inferiority. Front Neurol (2019) 10:695. doi: 10.3389/fneur.2019.00695 31379701 PMC6646514

[110] Nalbandian A Sehgal K Gupta A Madhavan MV McGroder C Stevens JS. Post-acute COVID-19 syndrome. Nat Med 2021 27:4 (2021) 27:601–15. doi: 10.1038/s41591-021-01283-z PMC8893149 33753937

[111] Chen C Haupert SR Zimmermann L Shi X Fritsche LG Mukherjee B. Global prevalence of post COVID-19 condition or long COVID: a meta-analysis and systematic review. J Infect Dis (2022) 226(9):1593–607. doi: 10.1093/INFDIS/JIAC136 PMC9047189 35429399

[112] Choutka J Jansari V Hornig M Iwasaki A. Unexplained post-acute infection syndromes. Nat Med (2022) 28:911–23. doi: 10.1038/s41591-022-01810-6 35585196

[113] Gold JE Okyay RA Licht WE Hurley DJ. Investigation of long COVID prevalence and its relationship to Epstein-Barr virus reactivation. Pathogens (Basel Switzerland) (2021) 10(6):763. doi: 10.3390/PATHOGENS10060763 34204243 PMC8233978

[114] Su Y Yuan D Chen DG Ng RH Wang K Choi J. Multiple early factors anticipate post-acute COVID-19 sequelae. Cell (2022) 185:881–895.e20. doi: 10.1016/J.CELL.2022.01.014/ATTACHMENT/B1FB9AE1-6442-495C-A1DF-6A5E16CA88FA/MMC7.XLSX 35216672 PMC8786632

[115] Bellucci G Rinaldi V Buscarinu MC Reniè R Bigi R Pellicciari G. Multiple sclerosis and SARS-CoV-2: has the interplay started? Front Immunol (2021) 12:755333. doi: 10.3389/fimmu.2021.755333 34646278 PMC8503550

